# Long-term Outcomes of Endovascular and Open Repair for Traumatic Thoracic Aortic Injury

**DOI:** 10.1001/jamanetworkopen.2018.7861

**Published:** 2019-02-08

**Authors:** Yu-Ting Cheng, Chi-Tung Cheng, Shang-Yu Wang, Victor Chien-Chia Wu, Pao-Hsien Chu, An-Hsun Chou, Ching-Chang Chen, Po-Jen Ko, Kuo-Sheng Liu, Shao-Wei Chen

**Affiliations:** 1 Division of Thoracic and Cardiovascular Surgery, Department of Surgery, Chang Gung Memorial Hospital, Linkou Medical Center, Chang Gung University, Taoyuan City, Taiwan; 2 Division of Trauma and Emergency Surgery, Department of Surgery, Chang Gung Memorial Hospital, Taoyuan, Taiwan; 3 Keelung Branch and Linkou Medical Center, Department of Cardiology, Chang Gung Memorial Hospital, Taoyuan City, Taiwan; 4 Linkou Medical Center, Department of Anesthesiology, Chang Gung Memorial Hospital, Chang Gung University, Taoyuan City, Taiwan; 5 Linkou Medical Center, Department of Neurosurgery, Chang Gung Memorial Hospital, Chang Gung University, Taoyuan City, Taiwan; 6 Graduate Institute of Clinical Medical Sciences, College of Medicine, Chang Gung University, Taoyuan City, Taiwan

## Abstract

**Importance:**

Thoracic endovascular aortic repair (TEVAR) has been adopted rapidly for treating traumatic thoracic aortic injury (TAI). The long-term durability and efficacy remain unknown.

**Objective:**

To determine the long-term outcomes of thoracic endovascular aortic repair and those of open repair (OR) for traumatic TAI.

**Design, Setting, Participants:**

This nationwide cohort study used Taiwan’s National Health Insurance Database to evaluate patients with traumatic TAI between January 1, 2004, and December 31, 2013. This single-payer National Health Insurance program covered more than 99.9% of the Taiwanese population. Those who received OR or TEVAR for TAI by propensity score matching were included. Data analysis was conducted in October 2017.

**Exposures:**

Thoracic endovascular aortic repair vs open repair.

**Main Outcomes and Measures:**

In-hospital outcomes, all-cause mortality, aorta reintervention, and stroke during follow-up.

**Results:**

Of the 287 patients (mean [SD] age, 41.66 [17.98] years; 80.5% male) who received OR or TEVAR for TAI, propensity score matching yielded 100 patients each in the OR and TEVAR groups. After propensity score matching, in-hospital mortality risk was significantly lower in the TEVAR group (9 [9.0%]) than in the OR group (27 [27.0%]; TEVAR vs open repair: odds ratio, 0.27; 95% CI, 0.12-0.60). With a mean (SD) follow-up of 2.80 (2.63) years, the proportion of survivors was 71.9% at 1 year, 70.8% at 2 years, 68.2% at 3 years, and 65.1% at 5 years in the OR group vs 88.9% at 1 year, 88.9% at 2 years, 88.9% at 3 years, and 88.9% at 5 years in the TEVAR group (log-rank test, *P* < .001). The TEVAR group had only 2 events (2%) of late reintervention and 1 event (1%) of late cerebrovascular accident. The proportion of freedom from reintervention was 100% at 1 year, 100% at 2 years, 100% at 3 years, and 100% at 5 years in the OR group vs 97.4% at 1 year, 97.4% at 2 years, 97.4% at 3 years, and 97.4% at 5 years in the TEVAR group (log-rank test, *P* = .18).

**Conclusion and Relevance:**

Compared with OR, TEVAR was associated with better long-term outcomes, mainly owing to lower mortality during the perioperative period. In addition, TEVAR was associated with a similar rate of survival and reintervention after hospital discharge.

## Key Points

**Question:**

What are the long-term durability and efficacy for traumatic thoracic aortic injury being treated by thoracic endovascular aortic repair ?

**Findings:**

In this cohort study of 287 patients in Taiwan, thoracic endovascular aortic repair was associated with better long-term outcomes, mainly owing to lower mortality during the perioperative period.

**Meaning:**

This study suggests that thoracic endovascular aortic repair may be more suitable than open repair for patients with traumatic thoracic aortic injury.

## Teaser

This cohort study uses propensity score matching to compare long-term outcomes of thoracic endovascular aortic repair vs open repair for the treatment of traumatic aortic injury in patients in Taiwan.

## Introduction

Traumatic thoracic aortic injury (TAI) is the second-leading cause of death among trauma patients.^1,2^ Baker et al^3^ and Trunkey^4^ defined the timing of trauma deaths as a trimodal distribution in urban environments in the United States; the distribution of death after traumatic injury can be characterized by 3 periods. Approximately 50% of deaths occur immediately (minutes), usually owing to severe neurological injury or frank rupture of a great vessel. The second peak of approximately 30% of deaths occurs early (hours), which is preventable death in modern trauma care—some call it the “golden hour.” The remaining 20% of deaths occurs late (weeks), secondary to sepsis or multiple organ failure.

The prehospital mortality rate for TAI is high, with 10% to 15% of patients surviving long enough to arrive at a hospital and receive treatment.^5^ Since 1959, open repair (OR) has been the standard treatment option for TAI. However, endovascular repair with a stent graft was introduced in the late 1990s. Endovascular repair of isolated descending thoracic aneurysms was approved by the US Food and Drug Administration in 2005.^6^ The indications of use were expanded to include traumatic aortic transection by the US Food and Drug Administration in 2012.

Although the use of thoracic endovascular aortic repair (TEVAR) for treating TAI is increasing,^7^ no relevant randomized clinical trials have been conducted thus far, to our knowledge. Therefore, no high-quality evidence has been available to determine the superiority of an endovascular approach to OR. The current clinical practice guideline regarding endovascular repair of traumatic TAI was published by the Society for Vascular Surgery in 2011.^8^ The guideline suggests that “endovascular repair be performed preferentially over open surgical repair or nonoperative management (grade 2, level C).”^8^ However, the strength of recommendation is weak and the quality of evidence is poor, both of which are based on a systematic review article by Murad et al.^9^ In addition, no long-term trauma patient follow-up has been used; one study even suggested up to 20% loss in the follow-up at first year.^10^

The use of endovascular repair has been adopted rapidly in the past decade as a treatment for TAI. However, the long-term durability and efficacy of TEVAR remain unknown. This is a critical topic for patients with TAI because this population is relatively younger and the medical follow-up compliance is poor. Therefore, we compared the long-term outcomes of TEVAR and OR for TAI in Taiwan by using a nationwide database.

## Methods

### Data Source

This study was executed using data from the National Health Insurance Research Database (NHIRD) in Taiwan. Since March 1995, Taiwan has implemented the single-payer National Health Insurance (NHI) program. More than 99.9% of the Taiwanese population is presently enrolled in the program. The NHIRD contains registration files and claim data for reimbursements from the NHI program. Patients’ identification numbers are encrypted and deidentified in the database. Diagnosis and procedures are coded according to the *International Classification of Diseases, Ninth Revision, Clinical Modification* (*ICD-9-CM*). The study was approved by the institutional review board of Chang Gung Memorial Hospital and informed consent was waived. This study followed the Strengthening the Reporting of Observational Studies in Epidemiology (STROBE) reporting guideline.

### Study Population

**Figure 1.**
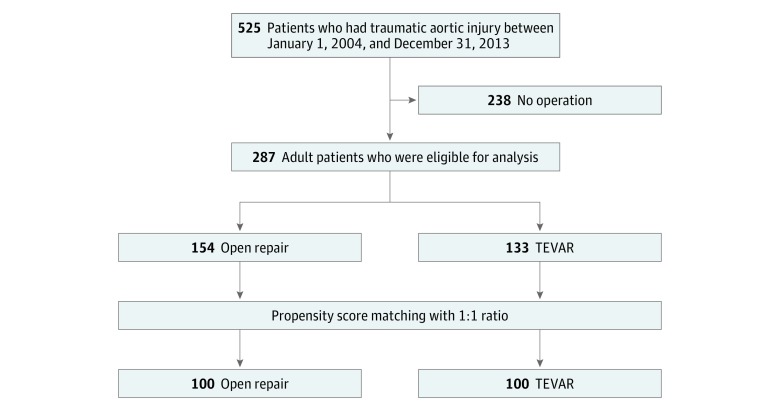
Enrollment of the Study Patients TEVAR indicates thoracic endovascular aortic repair.

The study cohort flowchart is shown in Figure 1. This is a retrospective nationwide cohort study. Patients hospitalized for newly diagnosed TAI (*ICD-9-CM* 901.0) between January 1, 2004, and December 31, 2013, were studied. Histories of TAI can be tracked to January 1, 1997, the beginning of the NHIRD. Patients with a Taiwanese NHI procedure code for thoracic aortic repair or replacement (69024, 69036, and 69037) were used for analysis. The patients were then divided into 2 groups: OR and TEVAR. The TEVAR group was identified by using *ICD-9-CM* codes 39.73 and 39.79, reconfirmed using the records of endovascular stent graft medical supplies codes. Patients younger than 18 years or with missing surgical characteristics were excluded (Figure 1).

### Comorbidities and Outcomes

Demographic data, clinical characteristics, and in-hospital outcomes were identified using *ICD-9-CM* or NHI procedure codes (eTable 1 in the Supplement). The comorbidities were detected by tracing patients’ hospitalization records before hospitalization. In-hospital outcomes included newly onset stroke, acute kidney injury, hemodialysis, pneumonia, sepsis, and gastrointestinal bleeding. The outcome measures were perioperative complications, in-hospital mortality, all-cause mortality, reintervention, and stroke during follow-up. The primary outcomes of mortality were determined by withdrawal from the NHI system.^11^ The accuracy of using *ICD-9-CM* codes, NHI procedure codes, and identification of the comorbidities of the NHIRD has been validated for patients with cardiovascular disease and patients who received cardiac surgery.^12,13,14^

### Propensity Score Matching

Through propensity score matching (PSM), we compared the OR and TEVAR groups. Each OR case was matched to 1 TEVAR case. The parameters used to calculate the propensity scores were age, sex, hospital level, comorbidities, associated organ injury, and fractures. Propensity score matching was conducted using the greedy nearest neighbor algorithm with a caliper width of 0.15 SD of the propensity score. An absolute standardized difference of less than 0.1 after matching was considered to indicate well-balanced matching variables between the groups.^15,16,17^ Propensity score matching yielded 100 cases each in the OR and TEVAR groups.

### Statistical Analysis

The demographic data and clinical characteristics of the 2 study groups were compared using the χ^2^ test for categorical data or *t* test for continuous variables. In-hospital outcomes included a binary event (ie, in-hospital mortality) and continuous variables (ie, ventilator days). The difference between the 2 groups was determined using logistic regression for binary events or linear regression for continuous variables. The results were presented as odds ratio (TEVAR vs open) or regression coefficient with the corresponding 95% CIs. To compare the all-cause mortality risk between the study groups, we constructed Kaplan-Meier survival curves along with log-rank tests. Data were analyzed using the R statistical package, version 3.3.4 (R Foundation), and PSM was performed using the MatchIt package for propensity matching.^18^ *P* < .05 was considered statistically significant. All *P* values were 2-sided. The date of the analysis was October 2017.

## Results

### Study Population Characteristics

Table 1 lists the baseline characteristics, comorbidities, and associated injuries in both study groups before and after PSM. We identified 287 patients (mean [SD] age, 41.66 [17.98] years; 80.5% male) who received OR or TEVAR for traumatic TAI between January 1, 2004, and December 31, 2011. The mean (SD) follow-up duration of these patients was 2.80 years (2.63) years. eTable 2 in the Supplement shows the percentage of reduction in bias for variables. eTable 3 in the Supplement shows the demographic characteristics and associated injuries of the unmatched and matched groups.

**Table 1.** Demographic Characteristics and Associated Injuries of the Open Repair and TEVAR Groups

Variable	Before Matching				After Matching			
	Open Repair (n = 154)	TEVAR (n = 133)	SMD (95% CI)	*P* Value	Open Repair (n = 100)	TEVAR (n = 100)	SMD (95% CI)	*P* Value
Age, mean (SD), y	40.68 (17.79)	42.79 (18.19)	0.117 (−0.115 to 0.349)	.32	41.95 (17.92)	41.83 (18.24)	0.007 (−0.270 to 0.284)	.96
Men, No. (%)	123 (79.9)	108 (81.2)	0.034 (−0.198 to 0.266)	.89	79 (79.0)	79 (79.0)	<0.001 (−0.277 to 0.277)	>.99
Medical center, No. (%)	54 (35.1)	34 (25.6)	0.208 (−0.025 to 0.440)	.11	31 (31.0)	27 (27.0)	0.088 (−0.189 to 0.366)	.64
Comorbidity, No. (%)								
Hypertension	18 (11.7)	21 (15.8)	0.119 (−0.113 to 0.352)	.40	13 (13.0)	13 (13.0)	<0.001 (−0.277 to 0.277)	>.99
Diabetes	13 (8.4)	10 (7.5)	0.034 (−0.198 to 0.266)	.95	8 (8.0)	8 (8.0)	<0.001 (−0.277 to 0.277)	>.99
CAD	6 (3.9)	7 (5.3)	0.065 (−0.178 to 0.286)	.79	4 (4.0)	4 (4.0)	<0.001 (−0.277 to 0.277)	>.99
COPD	2 (1.3)	1 (0.8)	0.054 (−0.178 to 0.286)	>.99	1 (1.0)	1 (1.0)	<0.001 (−0.277 to 0.277)	>.99
Associated injury, No. (%)								
Brain	24 (15.6)	18 (13.5)	0.058 (−0.174 to 0.290)	.75	16 (16.0)	15 (15.0)	0.028 (−0.250 to 0.305)	>.99
Chest	65 (42.2)	61 (45.9)	0.074 (−0.158 to 0.306)	.62	40 (40.0)	41 (41.0)	0.020 (−0.257 to 0.298)	>.99
Cardiac	7 (4.5)	3 (2.3)	0.127 (−0.106 to 0.359)	.46	4 (4.0)	3 (3.0)	0.054 (−0.223 to 0.332)	>.99
Liver	29 (18.8)	30 (22.6)	0.092 (−0.140 to 0.324)	.53	22 (22.0)	25 (25.0)	0.071 (−0.206 to 0.348)	.74
Gastrointestinal	12 (7.8)	20 (15.0)	0.229 (−0.003 to 0.462)	.08	11 (11.0)	13 (13.0)	0.062 (−0.216 to 0.339)	.83
Kidney	4 (2.6)	3 (2.3)	0.022 (−0.210 to 0.254)	>.99	2 (2.0)	2 (2.0)	<0.001 (−0.277 to 0.277)	>.99
Femur fracture	19 (12.3)	26 (19.5)	0.198 (−0.035 to 0.431)	.13	15 (15.0)	17 (17.0)	0.055 (−0.223 to 0.332)	.85
Pelvic fracture	12 (7.8)	19 (14.3)	0.208 (−0.024 to 0.441)	.12	10 (10.0)	9 (9.0)	0.034 (−0.243 to 0.311)	>.99
Spine fracture	8 (5.2)	15 (11.3)	0.223 (−0.010 to 0.455)	.09	7 (7.0)	6 (6.0)	0.041 (−0.237 to 0.318)	>.99

Abbreviations: CAD, coronary artery disease; COPD, chronic obstructive pulmonary disease; SMD, standardized mean difference; TEVAR, thoracic endovascular aortic repair.

### National Trend of TAI Treatment

**Figure 2.**
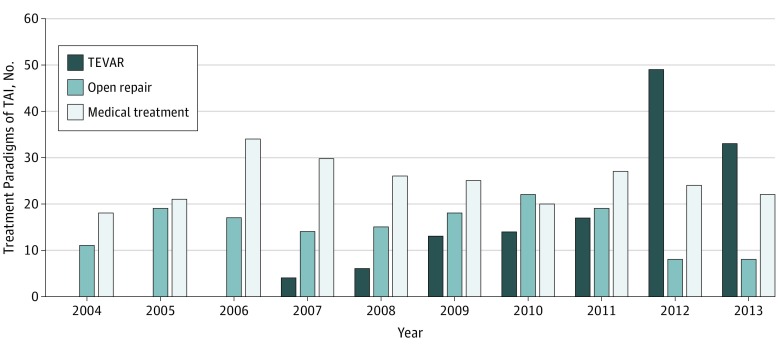
Changing Treatment Paradigms in Traumatic Thoracic Aortic Injury (TAI) in Taiwan TEVAR indicates thoracic endovascular aortic repair.

The change of treatment paradigms of TAI in Taiwan is presented in Figure 2. During the study, the percentage of nonsurgical treatment decreased. The proportion of patients who received OR was steady, but that of patients who received TEVAR increased over time. In the final 2 years of the study, TEVAR overtook OR as the mainstream TAI treatment in Taiwan.

### In-Hospital Mortality and Postoperative Complications

Table 2 lists the in-hospital mortality and postoperative complication rates. The TEVAR group had a significantly lower in-hospital mortality risk than the OR group (9 [9.0%] vs 27 [27.0%]; odds ratio, 0.27; 95% CI, 0.12-0.60; *P* = .002). Postoperative complications were common in patients with TAI. The risk of any morbidity was lower in the TEVAR group than in the OR group (12.0% vs 25.0%; odds ratio, 0.41; 95% CI, 0.19-0.87; *P* = .02). The risk of new hemodialysis was lower in the TEVAR group than in the OR group (4.0% vs 12.0%; odds ratio, 0.31; 95% CI, 0.10-0.98; *P* = .047). The risk of sepsis was also lower in the TEVAR group than in the OR group (2.0% vs 9.0%; odds ratio, 0.21; 95% CI, 0.04-0.98; *P* = .047). There were no significant differences in the risk of stroke, acute kidney injury, or pneumonia. The transfusion requirements including whole blood (mean [SD]: TEVAR, 0.98 [2.78] vs OR, 2.37 [8.01] units; *P* = .004), packed red blood cells (mean [SD]: TEVAR, 5.92 [8.56] vs OR, 12.79 [16.35] units; *P* < .001), and fresh frozen plasma (mean [SD]: TEVAR, 8.75 [19.20] vs OR, 15.24 [23.50] units; *P* < .001) were all significantly lower in the TEVAR group than in the OR group. The TEVAR group, compared with the OR group, required fewer ventilator days (mean [SD], 1.32 [1.40] vs 2.10 [2.83] days) and shorter intensive care unit lengths of stays (mean [SD], 1.53 [0.92] vs 1.88 [1.6] days) than the OR group; however, the difference was not significant for intensive care unit length of stay.

**Table 2.** Outcomes and Complications of Operations

Outcome and Complications	Open Repair, No. (%) (n = 100)	TEVAR, No. (%) (n = 100)	TEVAR vs Open Repair, Odds Ratio (95% CI)	*P* Value
Binary event				
Any morbidity	25 (25.0)	12 (12.0)	0.41 (0.19-0.87)	.02
Stroke	7 (7.0)	1 (1.0)	0.13 (0.02-1.11)	.06
New hemodialysis	12 (12.0)	4 (4.0)	0.31 (0.10-0.98)	.047
Pneumonia	8 (8.0)	4 (4.0)	0.48 (0.14-1.65)	.24
Sepsis	9 (9.0)	2 (2.0)	0.21 (0.04-0.98)	.047
GI bleeding	2 (2.0)	4 (4.0)	2.04 (0.37-11.41)	.42
Acute kidney injury	6 (6.0)	1 (1.0)	0.16 (0.02-1.34)	.09
In-hospital mortality	27 (27.0)	9 (9.0)	0.27 (0.12-0.60)	.002
Continuous variable, mean (SD)				
Blood transfusion				
Whole blood	2.37 (8.01)	0.98 (2.78)	NA	.004
Packed RBCs	12.79 (16.35)	5.92 (8.56)	NA	<.001
Fresh frozen plasma	15.24 (23.50)	8.75 (19.20)	NA	<.001
Ventilator day, d	2.10 (2.83)	1.32 (1.40)	NA	.047
ICU day, d	1.88 (1.68)	1.53 (0.92)	NA	.99
Medical expenditure, thousands, $^a^	21.7 (18.8)	23.4 (13.0)	NA	.01

Abbreviations: GI, gastrointestinal; ICU, intensive care unit; NA, not applicable; RBCs, red blood cells; TEVAR, thoracic endovascular aortic repair.

^a^ One US dollar equals 31 Taiwan dollars.

We also evaluated the risk factor of in-hospital mortality. The result demonstrated brain injury was a risk factor for in-hospital mortality (11.8% vs 29.2%; OR, 3.06, 95% CI, 1.46-6.43; *P* = .003).

### Mortality and Complication During the Follow-up Period

**Figure 3.**
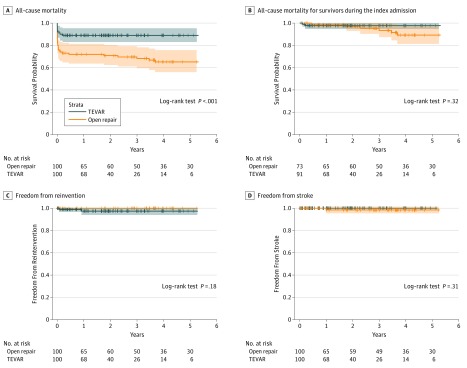
Kaplan-Meier Survival Curve of Follow-up Outcomes TEVAR indicates thoracic endovascular aortic repair.

The Kaplan-Meier survival plots for the differences between the TEVAR and OR groups are presented in Figure 3A (log-rank test, *P* < .001). The proportion of survivors was 71.9% at 1 year, 70.8% at 2 years, 68.2% at 3 years, and 65.1% at 5 years in the OR group vs 88.9% at 1 year, 88.9% at 2 years, 88.9% at 3 years, and 88.9% at 5 years in the TEVAR group. After excluding patients deceased during admission, the Kaplan-Meier survival plots (Figure 3B) were comparable (log-rank test, *P* = .32). The proportion of survivors was 98.6% at 1 year, 97.0% at 2 years, and 89.2% at 5 years in the OR group vs 97.8% at 1 year, 97.8% at 2 years, and 97.8% at 5 years in the TEVAR group. During the 6-year follow-up, 2 late reinterventions (2%) in the TEVAR group (Figure 3C) and 1 late cerebrovascular accident (1%) in the OR group (Figure 3D) were noted. The proportion of freedom from reintervention was 100% at 1 year, 100% at 2 years, 100% at 3 years, and 100% at 5 years in the OR group vs 97.4% at 1 year, 97.4% at 2 years, 97.4% at 3 years, and 97.4% at 5 years in the TEVAR group (log-rank test, *P* = .18). Reintervention rates and strokes did not significantly differ between the 2 groups.

## Discussion

Although the Society of Vascular Surgery guidelines recommend the use of endovascular repair over open surgical repair for traumatic TAI,^8^ the potential benefit and long-term durability and efficiency of this less invasive treatment remain uncertain. The current most comprehensive evidence is from a systemic review by Murad et al,^9^ which was based on 27 comparative observational nonrandomized studies. To our knowledge, no randomized clinical trial has evaluated this topic. Therefore, we organized the study using data from the Taiwan NHIRD. The database contains more than 99.9% of the population of Taiwan and records every medical procedure in Taiwan. To our knowledge, this is the first study to compare the long-term outcomes of operative treatment for patients with TAI who received TEVAR vs those of a matched OR control group. We demonstrated the better long-term outcome of TEVAR compared with that of OR regarding TAI management, mainly owing to decreased mortality in perioperative period. In addition, TEVAR was associated with similar rates of survival and reintervention after hospital discharge.

In-hospital mortality rates in the TEVAR and OR groups were significantly different (9.0% and 27.0%, respectively). A nationwide retrospective cohort study from Japan by Tagami et al^19^ also demonstrated that the in-hospital survival rate was higher in patients receiving TEVAR than in those receiving OR (15.8% vs 5.6%, respectively; log-rank χ^2^, 4.9; *P* = .03). Mousa et al^20^ also reported a significant difference for hospital mortality rates between TEVAR and OR (7.43% vs 14.61%, respectively; *P* = .009) from the National Inpatient Sample, containing a sample of 20% of the hospitals in the United States. In a meta-analysis, Tang et al^21^ demonstrated a lower 30-day mortality rate after TEVAR than after OR (7.6% vs 15.2%, respectively; *P* = .008). These results are consistent with our conclusion. After PSM, the TEVAR group had a significantly lower in-hospital mortality rate than did the OR group. The study’s reported in-hospital mortality rate was higher than that reported previously. This could be associated with the full reimbursement of stent graft costs. According to a medical center in Taiwan,^22^ 7.5% of patients with TAI had preoperation cardiopulmonary cerebral resuscitation before TEVAR.

Long-term follow-up with trauma patients is difficult. When the health of patients with trauma improves, the patients rarely return to previous clinics. Consequently, trauma patients are not followed up for a long duration. Kidane et al^10^ suggested the loss to follow-up at 1 year was 26.5% ± 31.6% after endovascular repair for traumatic TAI. Khashram et al^23^ presented the New Zealand national experience of TEVAR; although their median follow-up duration was 76.3 months, their sample size was small (only 88 patients). By using the NHIRD, we followed up with 100 patients who received TEVAR in Taiwan and demonstrated the favorable long-term outcomes in a PSM cohort that had received OR. The loss to follow-up rate in our study was 0. Because the NHI is comprehensive and affordable in Taiwan, patients seldom cancel their insurance policies, particularly after being diagnosed with major diseases or receiving major surgery.

The mortality of TEVAR treatment for TAI from Taiwan was relatively high (9.0%) compared with other studies from Japan or the United States.^19,24^ The use of TEVAR for TAI is fully reimbursed under the Taiwan NHI. Therefore, TEVAR is used for TAI treatment without delay and without consideration of the financial costs. No doubt, the NHI program has strict regulations regarding TEVAR reimbursements, which are granted to patients who only have computed tomography evidence of TAI, such as intramural hematoma, pseudoaneurysm, or frank aortic rupture. However, in critical patients such as those with massive hemothorax, periarrest, or even on return of spontaneous circulation patients after cardiac arrest, TEVAR might still be used by surgeons as a salvage procedure. There are no financial burdens in the surgeon’s mind. This could explain why reimbursement of stent grafts might lead to a higher mortality in Taiwan.

Reintervention is a major limitation of stent graft treatment. Studies have reported up to a 32% reintervention rate in endovascular-treated complicated aortic dissection disease.^25^ In particular, for the young patient population with TAI, long-term durability is critical. By using the NHIRD, we accessed data on every procedure at every hospital institute in Taiwan. In our study, 2 reinterventions (2.0%) by endovascular treatments in the TEVAR group occurred. Khashram et al^23^ suggested a 2.2% reintervention rate. In our study, the durability of TEVAR was similar to that of OR (log-rank test, *P* = .18).

### Limitations

This study had limitations. First, the major limitation of this study was that unmeasured confounders cannot be considered and adjusted. By using a national administration data set, specific details for clinical information such as trauma mechanism, computed tomography image, grade of traumatic aortic injury, aortic anatomy, and hemodynamic status, as well as procedure-related information such as aortic size, extent of repair, stent graft size, landing zone, and adjunctive endovascular procedure were all unavailable. The distribution of these unmeasured confounders could differ substantially in the 2 groups, which may induce a biased estimate of the treatment effect. However, the NHI has strict regulations on endovascular stent graft usage, potentially ensuring the proper use of endovascular stent grafts. Second, because the duration from TAI to surgery could not be calculated because of a limitation of the NHIRD database, the selection bias of stable and noncritical patients in the TEVAR group should be considered. Third, the TEVAR group and OR group are well balanced after PSM, but the matched group was different from the cases that were unmatched. Thus the comparison between matched groups cannot fully represent the whole population of patients with TAI. Fourth, misclassification and inaccurate *ICD-9* codes or database records could have occurred without detection. Despite these limitations, we believe our results still provide a substantial contribution to the analysis of long-term outcomes in patients with traumatic TAI.

## Conclusions

According to the present nationwide study, we used PSM to demonstrate the better long-term outcome of TEVAR compared with that of OR regarding TAI management, mainly owing to lower mortality in the perioperative period. In addition, TEVAR was associated with similar rates of survival and reintervention after hospital discharge. We recommend using TEVAR over OR for patients with TAI.

## Notes

Supplement. **eTable 1.** *ICD-9-CM* Code Used in the Current Study **eTable 2.** Percent Reduction in Bias for Variables **eTable 3.** Demographic Characteristics and Associated Injuries of the Unmatched and Matched Group
