# Supplementary material for: Long-term Outcomes of Endovascular and Open Repair for Traumatic Thoracic Aortic Injury
Source: JAMA Netw Open. 2019 Feb 8;2(2):e187861. doi: 10.1001/jamanetworkopen.2018.7861 (PMC6484615; doi:10.1001/jamanetworkopen.2018.7861)
Supplement: Supplement. — eTable 1. ICD-9-CM Code Used in the Current Study eTable 2. Percent Reduction in Bias for Variables eTable 3. Demographic Characteristics and Associated Injuries of the Unmatched and Matched Group [file jamanetwopen-2-e187861-s001.pdf]

Supplementary Online Content

Cheng Y-T, Cheng C-T, Wang S-Y, et al. Long-term outcomes of endovascular and open repair for traumatic thoracic aortic injury. *JAMA Netw Open*. 2019;2(2):e187861. doi:10.1001/jamanetworkopen.2018.7861

eTable 1. *ICD-9-CM* Code Used in the Current Study

eTable 2. Percent Reduction in Bias for Variables

eTable 3. Demographic Characteristics and Associated Injuries of the Unmatched and Matched Group

This supplementary material has been provided by the authors to give readers additional information about their work.

© 2019 Cheng Y-T et al. *JAMA Network Open*.

eTable 1. *ICD-9-CM* Code Used in the Current Study

Diagnosis	ICD-9-CM code
Hypertension	401, 402, 403, 404, 405
Diabetes mellitus	250
Coronary artery disease	410, 411, 412, 413, 414
Chronic obstructive pulmonary disease	491, 492, 496
Dialysis	585.xx (Catastrophic illness card)
Brain injury	850.3, 850.4, 850.5, 850.9, 851, 852, 853, 854, (800, 801, 802, 803, 804)
Chest injury	860.2, 860.3, 860.4, 860.5, 861.2, 861.3
Cardiac injury	861.0, 861.1
Liver injury	864
Gastrointestinal injury	863, 865
Kidney injury	866
Femur fracture	820, 821
Pelvic fracture	808
Spine fracture	805, 806
Stroke	430, 431, 432, 433, 434, 435, 436, 437
Pneumonia	480, 481, 482, 483, 484, 485, 486
Sepsis	038, 440.24, 590.10, 599.0, 682.6, 707.0, 728.86, 730.27, 785.4, 790.7, 996.62, 997.62
Gastrointestinal bleeding	530.21, 530.7, 530.82, 531, 532, 533, 534, 535, 537.83, 537.84, 578
Acute kidney injury	584, 634.3, 635.3, 636.3, 637.3, 638.3, 639.3, 669.3, 985.5
Ventilator	967.0, 967.1, 967.2

© 2019 Cheng Y-T et al. *JAMA Network Open*.

eTable 2. Percent Reduction in Bias for Variables

Characteristics	Percent of bias reduction (%)
Age, mean (SD), y	99.7
Men, No. (%)	100
Medical center, No. (%)	88.6
Comorbidity, No. (%)	
Hypertension	100
Diabetes mellitus	100
CAD	100
COPD	100
Associated Injury, No. (%)	
Brain	93.6
Chest	97.6
Cardiac	77.8
Liver	84.0
Gastrointestinal	74.4
Kidney	100
Femur fracture	83.7
Pelvic fracture	87.2
Spine fracture	80.8

Abbreviations: CAD, Coronary artery disease; COPD, chronic obstructive pulmonary disease

© 2019 Cheng Y-T et al. *JAMA Network Open*.

eTable 3. Demographic Characteristics and Associated Injuries of the Unmatched and Matched Group

Characteristics	Unmatched (N=87)	Matched (N=200)	SMD
Age, mean (SD), y	41.12(17.94)	41.89(18.03)	0.043
Men, No. (%)	73(83.9)	158(79.0)	0.127
Medical center, No. (%)	30(34.5)	58(29.0)	0.118
Comorbidity, No. (%)			
Hypertension	13(14.9)	26(13.0)	0.056
Diabetes mellitus	7(8.0)	16(8.0)	0.002
CAD	5(5.7)	8(4.0)	0.081
COPD	1(1.1)	2(1.0)	0.014
Associated Injury, No. (%)			
Brain	11(12.6)	31(15.5)	0.082
Chest	45(51.7)	81(40.5)	0.227
Cardiac	3(3.4)	7(3.5)	0.003
Liver	12(13.8)	47(23.5)	0.251
Gastrointestinal	8(9.2)	24(12.0)	0.091
Kidney	3(3.4)	4(2.0)	0.089
Femur fracture	13(14.9)	32(16.0)	0.029
Pelvic fracture	12(13.8)	19(9.5)	0.134
Spine fracture	10(11.5)	13(6.5)	0.175

Abbreviations: CAD, Coronary artery disease; COPD, chronic obstructive pulmonary disease.

© 2019 Cheng Y-T et al. *JAMA Network Open*.
